# Calcium release deficiency syndrome: an emerging ryanodinopathy

**DOI:** 10.3389/fmolb.2026.1810279

**Published:** 2026-05-15

**Authors:** Kaiyang Gao, Lan Tao, Xiaoqing Li, Baihe Li, Nike Li, Jinhong Wei

**Affiliations:** School of Medicine, Northwest University, Xi’an, China

**Keywords:** arrhythmias, calcium release deficiency syndrome, channelopathy, loss of function, ryanodine receptor

## Abstract

The cardiac ryanodine receptor (RyR2) is a critical calcium release channel essential for normal cardiac contraction. Dysregulation of RyR2 function is a key pathogenic factor in various cardiac diseases, notably arrhythmias and heart failure. The clinical spectrum of RyR2-related diseases (ryanodinopathy) has expanded beyond the well-established gain-of-function (GOF) mutations causing catecholaminergic polymorphic ventricular tachycardia (CPVT). Recent research has now delineated two additional distinct clinical entities: exon 3 deletion syndrome (E3DS), which presents with CPVT along with structural abnormalities, and calcium release deficiency syndrome (CRDS), caused by loss-of-function (LOF) mutations. CRDS is characterized by impaired calcium release from the sarcoplasmic reticulum and fundamentally differs from the classical CPVT phenotype, necessitating distinct approaches to its diagnosis, clinical management, and therapeutic intervention. This review provides a comprehensive overview of the current understanding of CRDS. We discuss its clinical manifestations, scrutinize the underlying molecular mechanisms, evaluate available diagnostic strategies, and explore potential therapeutic avenues. By synthesizing latest findings, this review aims to illuminate the complexities of RyR2-mediated pathologies and foster further progress in this evolving field.

## 1 Introduction

Calcium functions as a critical second messenger that exerts multifaceted and hierarchical control over cardiac function at both the organ and cellular levels. Its most fundamental role lies in triggering cardiac contraction. This process is central to excitation-contraction coupling (ECC): membrane depolarization prompts a minor influx of extracellular Ca^2+^ through L-type calcium channels, which in turn activates the release of a larger Ca^2+^ store from the sarcoplasmic reticulum (SR) via cardiac ryanodine receptor (RyR2). This concerted action generates a rapid surge in cytosolic Ca^2+^ concentration, known as the Ca^2+^ transient (Bers, 2002). The elevated cytosolic Ca^2+^ then binds to troponin C (TnC) on the myofilaments, inducing a conformational shift that initiates cross-bridge cycling and sarcomere shortening, thereby culminating in myocardial contraction.

For relaxation to occur, cytosolic Ca^2+^ must be rapidly removed. This is achieved primarily through its reuptake into the SR by the sarcoplasmic/endoplasmic reticulum calcium ATPase (SERCA) and its extrusion from the cell via the Na^+^-Ca^2+^ exchanger (NCX) (Bers, 2002; Eisner et al., 2017). The consequent decline in cytosolic Ca^2+^ prompts its dissociation from TnC, leading to myocardial relaxation and the completion of the contraction-relaxation cycle. The precision of this cyclical Ca^2+^ handling is a primary determinant of both the force of cardiac contraction and the efficiency of diastolic relaxation.

Furthermore, Ca^2+^ serves as a key modulator of cardiac electrical activity. The influx of Ca^2+^ through L-type calcium channel (LTCC) dynamically balances the efflux of K^+^, a process essential for the formation and maintenance of the action potential plateau in cardiomyocytes. This delicate balance ensures the proper duration of the action potential plateau and timely repolarization, which is essential for preventing afterdepolarizations and allowing complete relaxation and subsequent ventricular filling during diastole (Tonko and Lambiase, 2024; Zhong and Karma, 2024). Aside from working myocardium, the depolarization of pacemaker cells in the sinoatrial node is critically dependent on both T-type and L-type calcium channels. The inward Ca^2+^ current during phase-4 drives the slow diastolic depolarization, which is fundamental to the generation and modulation of the normal rhythm of the heart (Namekata et al., 2022).

In summary, through its regulation of myocardial contraction, electrical activity, and intracellular signaling pathways, Ca^2+^ emerges as a central orchestrator of cardiac function. The precise maintenance of Ca^2+^ homeostasis is, therefore, a critical mechanism underpinning normal cardiac pumping efficiency, electrical rhythm stability, and metabolic adaptation.

RyR2 is a Ca^2+^ release channel protein embedded in the SR membrane. Composed of four identical subunits and interacting with various modulators to form an intricate regulatory supercomplex (Peng et al., 2016; Gong et al., 2019; Marks, 2023; Hadiatullah et al., 2025), RyR2 opens in response to extracellular Ca^2+^ influx. This mediates the release of Ca^2+^ from the SR into the cytoplasm, a pivotal step in cardiac ECC (Bers, 2002). Beyond its physiological role, RyR2 dysfunction is implicated in various cardiac pathologies, including arrhythmias and heart failure (HF) (Belevych et al., 2013; Zaffran et al., 2023; Miotto et al., 2024). Recent advances have further expanded the spectrum of RyR2-related diseases, demonstrating that RyR2 dysfunction also contributes to the pathogenesis of dilated cardiomyopathy (DCM) (Xie et al., 2026), and is also implicated in metabolic heart diseases such as diabetes and prediabetes (Federico et al., 2017; Tow et al., 2022), thus broadening the clinical relevance of this calcium channel beyond inherited arrhythmias.

The association between RyR2 dysfunction and Ca^2+^ cycling disorder has been uncovered previously (Keefe et al., 2023). Catecholaminergic polymorphic ventricular tachycardia (CPVT) is a typical ventricular arrhythmia (VA) linked to RyR2 dysfunction. The most common form, CPVT1, is caused by RyR2 gain-of-function (GOF) mutations (Priori et al., 2001). CPVT can also result from mutations in RyR2-associated proteins such as calsequestrin 2 (CASQ2) and calmodulin (CaM) (Priori et al., 2002; Crotti et al., 2023). The estimated prevalence of classical CPVT is probably 1:10,000 (Priori et al., 2021). So far, CPVT has been characterized by sudden cardiac death (SCD) in the absence of structural heart disease, as well as bidirectional and/or polymorphic ventricular tachycardia (VT) that can be reproducibly induced by exercise stress testing (EST) (Priori and Chen, 2011). Notably, exon 3 deletion syndrome (E3DS) is an established, distinct entity of RyR2 channelopathy (ryanodinopathy), where CPVT with additional features/phenotypes e.g., dilated cardiomyopathy, atrial standstill has been described (Bhuiyan et al., 2007; Steinberg et al., 2023), mechanism of which is almost similar to CPVT alone, but in E3DS exon 3 deletion destabilizes the N-terminal structure of the RyR2 channel, facilitating premature pore opening and diastolic calcium leak for a longer period (Lobo et al., 2011). The incidence of E3DS is approximately 1:100,000 (Marjamaa et al., 2009; Steinberg et al., 2023).

**FIGURE 1:**
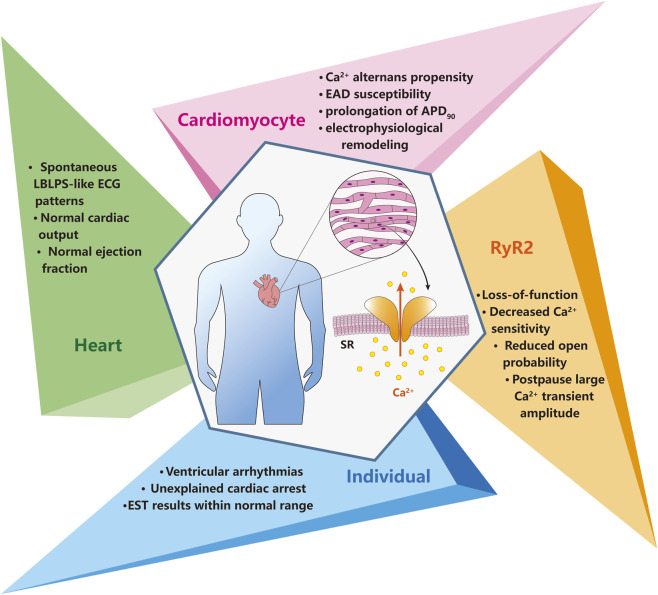
Apprehending CRDS through varying lenses. The four sections in the figure respectively illustrate the currently known pathological characteristics of CRDS, exhibited across various levels: the individual, the intact heart, the cardiac myocyte, and the RyR2 single channel. EST, exercise stress testing. LBLPS, long-burst, long-pause and short-coupled. ECG, electrocardiogram. EAD, early afterdepolarization. SR, sarcoplasmic reticulum.

However, an increasing number of patients harbouring RyR2 mutations with negative EST were reported (Roston et al., 2017b; Santiago and Priori, 2022). *In vitro* functional studies have identified that the overwhelming majority of these “atypical” CPVT individuals exhibited RyR2 loss-of-function (LOF) mutations, a condition termed cardiac ryanodine receptor calcium release deficiency syndrome (CRDS) (Sun et al., 2021). Accordingly, CRDS is regarded as an emerging ryanodinopathy entity distinguished from CPVT (Figure 1). Here, we will summarize the current understanding of the manifestation, mechanism, diagnosis and therapies of CRDS.

## 2 Clinical manifestations and genetic mapping of CRDS

Genetic research and testing conducted on CRDS patients’ families suggested that mutations in the *RYR2* gene (encoding RyR2) may be responsible for the inheritance of CRDS (Roston et al., 2017a; 2022; Sun et al., 2021). All the probands in these families suffered from SCD or aborted SCD (aSCD) and had rare RyR2 mutations functionally identified as RyR2 LOF variants. Notably, a subset of CRDS patients exhibited more complex life-threatening syndromes, including short-coupled variant of torsades de pointes (scTdP) and cardiomyopathy (Fujii et al., 2017). Despite the consensus that a disease entity should not be defined by DNA sequencing without concern for the phenotype in clinical practice, the gold standard for identifying CRDS individuals has been *RYR2* gene mutation detection thus far, since they typically do not exhibit clearly distinguishable phenotypes on routine clinical testing. Nevertheless, the unique clinical and molecular features associated with CRDS still indicate that it is a distinct inherited arrhythmogenic syndrome from the known ones.

**FIGURE 2:**
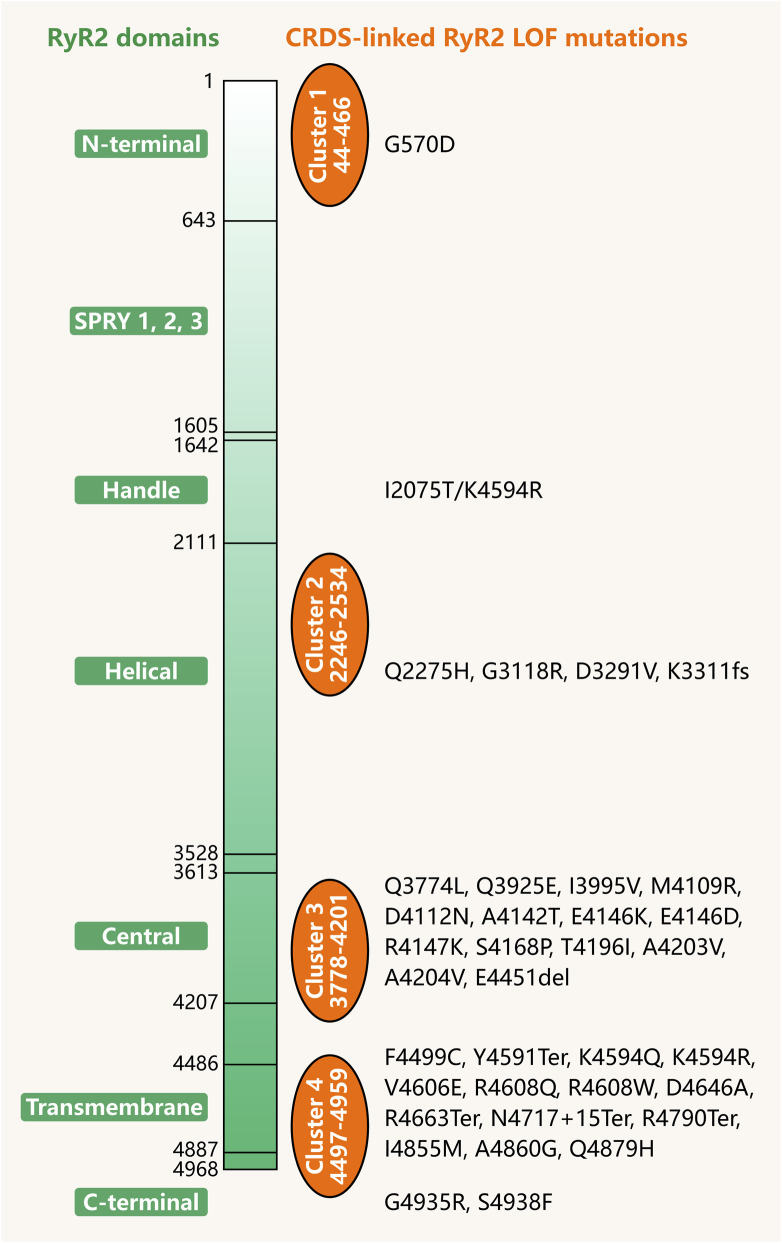
The identified CRDS mutant variants and their localization of mutation sites on RyR2. The structural domain division of RyR2 is shown on the left of the number axis, and the reported CRDS RyR2 LOF variants are displayed on the right. Orange circles indicate hotspots clusters of RyR2 mutations.

The genetic mutations accounting for CRDS were located in the *RYR2* gene, and have been functionally evaluated to result in RyR2 LOF (Priori et al., 2002; Jiang et al., 2007; Medeiros-Domingo et al., 2009; Nof et al., 2011; Tester et al., 2012; 2020; Paech et al., 2014; Kron et al., 2015; Fujii et al., 2017; Roston et al., 2017a; 2022; Blancard et al., 2021; Li et al., 2021; Shauer et al., 2021; Sun et al., 2021; Zhong et al., 2021; Hirose et al., 2022; Hopton et al., 2022; Ormerod et al., 2022; Tian et al., 2023). For a summary of the documented CRDS-associated RyR2 LOF mutations, see Table 1. The hotspot regions of RyR2 mutations linked to CRDS were concentrated in the central domain and the transmembrane domain located at the C-terminal (Figure 2), both of which are essential to the gating of the RyR2 pore and its activity (Peng et al., 2016; Uchinoumi et al., 2025).

**TABLE 1:** Clinical features and genetic analysis of CRDS RyR2 LOF cases. The literature cited in square brackets indicates the origin of the cases, which were subsequently confirmed as CRDS RyR2 LOF variants in later studies.

CRDS-linked RyR2 LOF genotypes	Mutation-located RyR2 domains	Patient phenotypes	References
A4860G*	Transmembrane domain	Syncope, cIVF	(Jiang et al., 2007; Priori et al., 2002)]
I4855M*	Transmembrane domain	SCA, SCD, LVNC	Roston et al. (2017a)
*RYR2-DUP*	Promoter region and exons 1–4	Syncope, SCA, SCD	Tester et al. (2020)
Q3774L	Central domain	aSCD	Sun et al. (2021)
I3995V	Central domain	Syncope, SCD, aSCD	
D4112N	Central domain	SCD, aSCD	
T4196I	Central domain	Syncope, SCD, aSCD	
D4646A*	Transmembrane domain	SCD, aSCD	
Q4879H	Transmembrane domain	aSCD	
I2075T/K4594R	Handle domain and transmembrane domain	Seizures, SCD, aSCD, IVF	[(Paech et al., 2014)]
G3118R	Helical domain	SCA, SCD, aSCD, VF	Shauer et al. (2021)
E4146K	Central domain	SCA, SCD	(Zhong et al., 2021; Tester et al., 2012)]
G4935R	C-terminal domain	Seizures, SCD	
D3291V	Helical domain	Syncope, SCD, aSCD	Blancard et al. (2021)
G570D	N-terminal domain	SCA, SCD	Li et al. (2021)
Q3925E	Central domain	SCD	[(Medeiros-Domingo et al., 2009)]
M4109R	Central domain	SCA, SCD, VF	[(Nof et al., 2011)]
R4147K	Central domain	Syncope, SCA, SCD	
A4203V	Central domain	Seizures, IVF	
A4204V	Central domain	SCA, VF	[(Kron et al., 2015)]
A4142T	Central domain	SCD, aSCD	Ormerod et al. (2022)
Q2275H	Helical domain	Syncope, SCA, SVT	Roston et al. (2022)
E4451del	Central domain	SCD, IRBBB	
F4499C	Transmembrane domain	Syncope, SCA, AF	
V4606E	Transmembrane domain	Syncope, seizures, SVT, AT, HF, DCM	
R4608Q	Transmembrane domain	Palpitations, syncope, SCA, SCD	
R4608W	Transmembrane domain	SCA	
E4146D	Central domain	Syncope, LQTS, scPVC, VF	Hirose et al. (2022)
S4168P	Central domain	LQTS, bradycardia	
K4594Q	Transmembrane domain	Syncope, LQTS	
S4938F	C-terminal domain	Syncope, scTdP, VF due to PVCs	[(Fujii et al., 2017)]
Y4591Ter	C-terminal truncating	SCA, NSVT	Tian et al. (2023)
R4663Ter	C-terminal truncating	Seizures, cognitive impairment	
N4717 + 15Ter	C-terminal truncating	SCD, syncope, bradycardia	
R4790Ter	C-terminal truncating	SCA, syncope	Hopton et al. (2022)
K3311fs	Frameshift mutation	VAs	ClinVar ID: 3065287

*Mutations for which mouse models have been generated.

AF, atrial fibrillation; aSCD, aborted sudden cardiac death; AT, atrial tachycardia; cIVF, catecholaminergic idiopathic ventricular fibrillation; DCM, dilated cardiomyopathy; IRBBB, incomplete right bundle branch block; IVF, idiopathic ventricular fibrillation; LQTS, long-QT, syndrome; LVNC, left ventricular non-compaction; NSVT, non-sustained Ventricular Tachycardia; PVC, premature ventricular complex. *RYR2-DUP*, *RYR2* homozygous multiexon duplication. SCA, sudden cardiac arrest; SCD, sudden cardiac death; scPVC, short coupled premature ventricular contract. scTdP, short-coupled variant of torsades de pointes. SVT, supraventricular tachycardia; VA, ventricular arrhythmia; VF, ventricular fibrillation.

EST for CRDS pedigree members, whether the probands or their affected relatives, showed normal outcomes or merely isolated PVCs (Li et al., 2021; Sun et al., 2021; Ormerod et al., 2022; Roston et al., 2022). In addition, there were no abnormalities in the cardiac structure or ejection fraction of patients with CRDS (Sun et al., 2021; Roston et al., 2022). Given the small number of cases associated with CRDS and the heterogeneity of presentation across patients (Hirose et al., 2022; Roston et al., 2022), larger clinical studies are urgently needed to provide better treatment and management strategies for patients with CRDS. Furthermore, prognostic assessment and follow-up of patients with CRDS are crucial for timely detection and management of possible arrhythmias and other complications.

## 3 CRDS and CPVT: a systematic comparison

The most classic RyR2-ryanodinopathy is CPVT, characterized by polymorphic VT induced by excitation of the sympathoadrenal system. The majority of CPVT patients experienced syncope or cardiac arrest induced by exercise or emotional stress with onset in childhood or adolescence. CPVT phenotypes, however, were seldom inducible by programmed fast pacing electrical stimulation during electrophysiological examination, suggesting that this arrhythmia is dependent on catecholamines rather than accelerated heart rates alone (Chang et al., 2025).

EST, typically performed using a bicycle ergometer or treadmill, is a well-established tool for detecting exercise-induced arrhythmias. In the context of ryanodinopathy, EST plays a fundamental diagnostic role: a key criterion for CPVT is the reproducible induction of bidirectional or polymorphic VT during EST (Chang et al., 2025). However, the EST results of CRDS patients are mostly negative, meaning that EST cannot reliably induce arrhythmias in individuals with CRDS. Another difference from CPVT patients is the lack of a significant effect of adrenergic stimulation on inducing arrhythmic episodes in CRDS individuals (Roston et al., 2022; Zheng et al., 2022), which may be attributed to the LOF nature of RyR2 mutations, thereby attenuating the response to adrenergic stimulation.

The genetic basis of type 1 CPVT has been established as RyR2 GOF mutations, and the underlying arrhythmogenic mechanisms have been extensively studied, forming a well-established theoretical framework. RyR2 GOF mutations increase the propensity for spontaneous Ca^2+^ release from the SR during diastole in cardiomyocytes, activating the Na^+^/Ca^2+^ exchanger (NCX) to clear cytosolic Ca^2+^. This generates an inward current, thereby inducing delayed afterdepolarizations (DADs). Once DADs reach the threshold potential, they can trigger new action potentials, leading to triggered activity that ultimately progresses to malignant ventricular arrhythmias (VAs). Therefore, DADs induced by spontaneous Ca^2+^ release are recognized as the primary cellular electrophysiological basis for arrhythmogenesis in CPVT (Priori and Chen, 2011).

In terms of channel function, CRDS RyR2 LOF mutations suppressed the propensity for spontaneous and caffeine-induced Ca^2+^ release, reduced the sensitivity of RyR2 channels to Ca^2+^ concentration, but conversely facilitated Ca^2+^ alternans and prolonged the refractoriness of Ca^2+^ transients in intact hearts (Jiang et al., 2007; Zhong et al., 2016; 2021; Sun et al., 2021; Ormerod et al., 2022). For a comparison of CPVT and CRDS, see Table 2. The molecular pathogenic mechanisms of CRDS will be discussed in detail in Section 4.

**TABLE 2:** Comparison between CRDS and CPVT.

Feature	CPVT	CRDS
Clinical triggers	• Exercise or emotional stress (sympathoadrenal activation) • Reproducibly triggers syncope or cardiac arrest, often in childhood/adolescence	• Not clearly associated with adrenergic stimulation • Patients may present with SCD or aborted SCD without typical exercise-induced syncope
EST and ECG findings	• EST reliably induces bidirectional or polymorphic ventricular VT • Hallmark ECG feature during EST is reproducible VT	• EST mostly negative (normal or only isolated PVCs) • No reproducible induction of complex arrhythmias
Molecular mechanism	RyR2 GOF mutations (CPVT1) → increased propensity for diastolic spontaneous Ca2+ release from SR → activation of NCX → inward current →DADs → triggered activity → VT	RyR2 LOF mutations → reduced spontaneous and caffeine-induced Ca2+ release, decreased Ca2+ sensitivity of RyR2, but enhanced Ca2+ alternans propensity

EST, exercise stress test; SCD, sudden cardiac death; VT, ventricular tachycardia; DAD, delayed afterdepolarization.

## 4 Molecular pathogenic mechanisms of CRDS

The occurrence of CRDS is attributed to RyR2 LOF mutations, which reduce Ca^2+^ release from the SR during systole. This primary defect in RyR2 function serves as the fundamental basis for all subsequent alterations in membrane potential and ion currents discussed in this section. Every change in membrane potential and ion flux ultimately stems from, and is secondary to, the impaired RyR2-mediated Ca^2+^ release. The diminished Ca^2+^ release has two major consequences. First, it weakens Ca^2+^-dependent inactivation (CDI) of LTCC. Under normal conditions, the systolic Ca^2+^ release triggers CDI, which helps terminate I_CaL_. In CRDS, the reduced Ca^2+^ release relieves this negative feedback, leading to increased I_CaL_ amplitude (Sun et al., 2021). The enhanced I_CaL_ prolongs the action potential duration (APD) and creates a substrate for early afterdepolarizations (EADs). Second, the impaired Ca^2+^ release function of RyR2 leads to an excessive Ca^2+^ storage in the SR. When the activation threshold for store-overload-induced Ca^2+^ release (SOICR) is reached, the RyR2s open spontaneously and collectively, bringing about a drastic Ca^2+^ release within a single heartbeat. Under these circumstances, NCX engages in the removal of excess cytosolic Ca^2+^, further elevating membrane potential and elongating APD (Jiang et al., 2007; Zhao et al., 2015). It is important to distinguish these baseline alterations from triggered events. The large Ca^2+^ transients that precede VAs are typically provoked by specific challenges, which cause transient SR Ca^2+^ overload, as discussed in Section 5. In summary, EAD is the initial anomalous electrophysiological event in CRDS.

### 4.1 Ca^2+^ alternans and arrhythmogenesis

Ca^2+^ alternans can be taken to refer to abnormal, periodic alternations in intracellular Ca^2+^ concentration within cardiac myocytes throughout the cardiac cycle, which serves not merely as an indicator of dysregulated Ca^2+^ handling, but also as a harbinger of impending heart disease (Qu and Weiss, 2023). The emergence of Ca^2+^ alternans is robustly linked to serious pathological conditions, such as heart failure and myocardial ischemia (Varró et al., 2021). Functional studies have shown an increased tendency for Ca^2+^ alternans in intact hearts expressing RyR2 LOF mutations compared to RyR2 WT (Zhong et al., 2016; Sun et al., 2021). By promoting the instability of membrane potential during repolarization and disrupting the normal electrical activity of cardiomyocytes, Ca^2+^ alternans can trigger EADs and, subsequently, arrhythmias.

EADs are transient voltage oscillations that occur during the plateau or repolarization phase of the action potential, before full repolarization is achieved. When EADs reach threshold potential, they can trigger premature action potentials, giving rise to triggered activity, a focal arrhythmia mechanism (Wit, 2018). While EADs themselves serve as triggers, the progression to sustained arrhythmias such as VT or ventricular fibrillation (VF) often requires a vulnerable tissue substrate. For instance, spatial heterogeneity in repolarization created by EAD-mediated APD prolongation can establish conduction blocks and reentrant circuits (Stein et al., 2025). Thus, in the setting of CRDS, EADs may initiate premature beats that, in the presence of a susceptible substrate, degenerate into life-threatening arrhythmias and SCD.

### 4.2 L-type Ca^2+^ channel

**FIGURE 3:**
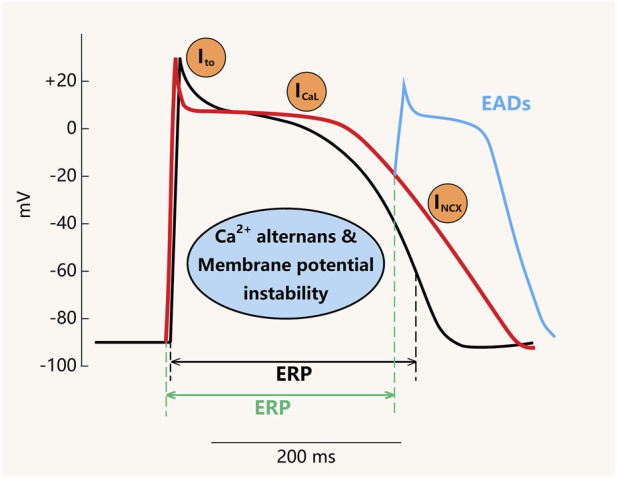
Changes in the action potential of cardiomyocytes in CRDS RyR2 LOF conditions. The black curve shows the physiological APD of cardiomyocytes, while the red curve depicts the alterations in membrane potential attributed to CRDS RyR2 LOF. The orange regions show the enhanced process, and the blue regions indicate the occurrence of EADs. ERP, effective refractory period. I_to_, transient outward potassium current. I_CaL_, L-type calcium current. I_NCX_, sodium-calcium exchange current. EAD, early afterdepolarization.

In CRDS, arrhythmogenesis stems from malfunctioning Ca^2+^ fluxes due to RyR2 LOF mutations (Steinberg et al., 2023). Owing to CRDS RyR2 LOF mutations followed by reduced SR Ca^2+^ release, cytosolic Ca^2+^ concentration becomes inadequate compared to that in RyR2 WT myocytes. As a consequence, LTCC is required to undertake a greater amount of Ca^2+^ influx to ensure calcium homeostasis and effective cardiac contractile function, thereby prolonging the action potential duration and increasing electrical instability during repolarization (Jiang et al., 2007; Zhao et al., 2015). However, the expression of LTCC (Cav1.2) is not significantly altered in RyR2 LOF variants. Functional studies have shown that I_CaL_ current density is enhanced in RyR2 LOF cardiomyocytes (Sun et al., 2021), contributing to action potential prolongation and arrhythmogenesis, although the efficiency of ECC remains reduced due to impaired RyR2-mediated Ca^2+^ release (Zhao et al., 2015). The prolonged action potential duration predisposes the cells to phase-2 EADs, which may propagate into triggered activity upon termination of the effective refractory period (ERP) (Figure 3). It is worth noting that the role of LTCC can only partially compensate for the lack of intracellular Ca^2+^ release. In severe cases of RyR2 dysfunction, the amount of Ca^2+^ inflow via LTCC cannot meet the physiological demand for contraction, which is a precursor of mutation-induced heart failure (Belevych et al., 2013).

### 4.3 Na^+^/Ca^2+^ exchanger

Also of interest is the pivotal role of NCX in calcium homeostasis. The dual-directional-operational NCX serves as an efficient Ca^2+^ extrusion system in cardiac myocytes. Returning to the source of RyR, ryanodine alkaloid is capable of locking the RyR channels in a sub-conductance state, resulting in sluggish and extended Ca^2+^ leakage from the SR, ultimately leading to macroscopic muscle paralysis. Curiously, ryanodine alkaloid elicits rigid paralysis in skeletal muscles, whereas cardiac muscles exhibit flaccid paralysis in response to the compound (Fill and Copello, 2002). This contrasting response highlights the unique Ca^2+^ handling strategy in the heart. The excessive Ca^2+^ leak via RyR after exposure to ryanodine alkaloid exceeds the reuptake capacity of SERCA, resulting in the accumulation of cytosolic Ca^2+^, which is the cause of rigid paralysis in skeletal muscles. In contrast, the presence of NCX in cardiomyocytes has tremendous potential to remove Ca^2+^ from the intracellular to the extracellular side, and therefore the calcium pool (referring to SR luminal [Ca^2+^]) will be exhausted over time. As a result, cardiac muscles are trapped in flaccid paralysis, since there is not enough intracellular Ca^2+^ ([Ca^2+^]_i_) available for systole anymore (Scranton et al., 2024).

Although CRDS inhibits SOICR and increases activation threshold for spontaneous Ca^2+^ release, there is no significant change in SR Ca^2+^ store capacity (Li et al., 2021; Sun et al., 2021; Ormerod et al., 2022). In other words, the initiation of RyR2-mediated Ca^2+^ release is simply more difficult rather than eliminated under CRDS. Paradoxically, when SOICR does occur, the magnitude of Ca^2+^ release becomes more pronounced due to an increased difference between the activation and termination thresholds of SOICR, which is elevated in CRDS-associated mutants (Ormerod et al., 2022). This increased difference between the activation and termination thresholds means that, when SOICR is triggered spontaneously, the resulting release event is more robust, producing larger and more widespread cytosolic Ca^2+^ elevations. These stochastic Ca^2+^ release events can activate NCX and generate depolarizing currents, potentially contributing to arrhythmic risk. To maintain Ca^2+^ electrochemical equilibrium, NCX operates in forward mode (3 Na^+^ influx for 1 Ca^2+^ efflux) at this moment. Similar to the multiple effects of LTCC, the inward current from NCX activation (I_NCX_) also leads to both membrane depolarization and APD prolongation. In CRDS, these systolic NCX activities increase the risk of phase-3 EADs, as evidenced by observable lengthening of APD_90_ (APD at 90% repolarization) (Sun et al., 2021). This mechanism is fundamentally different from that underlying CPVT, where diastolic Ca^2+^ waves activate NCX to generate DADs.

Taken together, although LTCC and NCX mediate Ca^2+^ fluxes in opposite directions, both generate inward currents (I_CaL_ and I_NCX_) from the plateau phase onward, thereby increasing the net inward current and promoting a substrate for EADs (Kettlewell et al., 2019; Lotteau et al., 2021). Furthermore, given that EADs occur during a period when membrane potential is not fully repolarized, ion channels other than mediating Ca^2+^ flux, particularly voltage-gated Na^+^ and K^+^ channels subjected to electrophysiological remodeling in CRDS, may also influence the substrate for EADs (Figure 3).

### 4.4 Na^+^ and K^+^ channels

Electrophysiological studies have shown variations in the Na^+^ current (I_Na_) and the transient outward K^+^ current (I_to_) in RyR2-D4646A^+/−^ variants of a confirmed CRDS genotype, compared to those observed in RyR2 WT. Specifically, I_to_ density of RyR2-D4646A^+/−^ cardiomyocytes was increased, and I_Na_ exhibited a hyperpolarizing shift in its voltage-dependent activation (i.e., activation at more negative potentials) (Sun et al., 2021). Although this shift does not imply earlier activation during a normal action potential, it may modulate I_Na_ availability during the upstroke. Together with the enhanced I_to_, these changes promote faster early repolarization, leading to a shortened APD_50_. The abbreviated APD_50_ may modestly accelerate Na^+^ channel recovery from inactivation, potentially contributing to the observed reduction in the ERP/APD ratio, although this effect is likely minor compared to other electrophysiological changes in CRDS. In summary, the increase in I_CaL_ and I_NCX_ activity is responsible for the prolongation of APD_90_, while the shortening of APD_50_ (APD at 50% repolarization) observed in CRDS models is associated with alterations in I_to_ and I_Na_, though the precise contribution of I_Na_ remodeling to early repolarization remains to be fully defined (Singh and Sanghvi, 2024; Zhou et al., 2024; Zheng et al., 2025). Paradoxically, the prolongation of repolarization observed in CRDS may represent a compensatory adaptation to RyR2 LOF. This extended repolarization phase may act through at least two parallel mechanisms: 1) it provides a longer temporal window for CICR through the impaired RyR2 channels, increasing the opportunity for release; and 2) it may reduce net Ca^2+^ extrusion via forward-mode NCX, thereby enhancing SR Ca^2+^ content and partially restoring releasable Ca^2+^ stores. Both mechanisms would help maintain essential ECC and preserve Ca^2+^ homeostasis despite the primary release defect. However, excessive APD prolongation also increases the risk of afterdepolarizations, tipping the balance from adaptation toward arrhythmogenesis.

When CRDS patients suffer from localized excessive Ca^2+^ influx during phase-2 of the APD, the relative magnitude of Ca^2+^ influx surpasses the K^+^ efflux at these specific sites. As a result, a depolarization event ensues, originating in phase-2 and potentially persisting into phase-3, altering the electrical activity of cardiac myocytes. Additionally, premature activation and inactivation of Na^+^ channels may lead to early termination of ERP, either in phase-2 or phase-3. Therefore, once the two events occur concurrently, there is a risk that the termination of ERP may be followed by the occurrence of EADs in either phase-2 or phase-3 (Figure 3).

### 4.5 Molecular basis of RyR2 LOF mutations

The mutation sites associated with CRDS RyR2 LOF are located within multiple RyR2 domains, all of which are acknowledged to be critical for the “open and shut” function of RyR2 (Priori et al., 2002; Jiang et al., 2007; Medeiros-Domingo et al., 2009; Nof et al., 2011; Tester et al., 2012; 2020; Paech et al., 2014; Kron et al., 2015; Fujii et al., 2017; Roston et al., 2017a; 2022; Blancard et al., 2021; Li et al., 2021; Shauer et al., 2021; Sun et al., 2021; Zhong et al., 2021; Hirose et al., 2022; Hopton et al., 2022; Ormerod et al., 2022; Tian et al., 2023). Indeed, these mutations apparently result in impaired RyR2 Ca^2+^ release function, but it merits consideration why they lead to RyR2 LOF. One hypothesis is that the mutation may directly alter the gating properties of RyR2 by destabilizing the spatial and charge distributions near the site. Alternatively, the mutation may indirectly affect the activity of RyR2 by influencing the binding state of various RyR2 regulators in the vicinity of the mutation site. Bringing to light the underlying mechanism of RyR2 LOF due to mutation needs detailed investigations into structural consequences of specific mutations in RyR2, interactions between mutated RyR2 and its modulators, and exploration of how multifarious mutations perturb RyR2 regulatory pathways. Understanding the molecular basis of RyR2 LOF would facilitate recognition of the clinical heterogeneity of CRDS.

## 5 Diagnosis of CRDS

**FIGURE 4:**
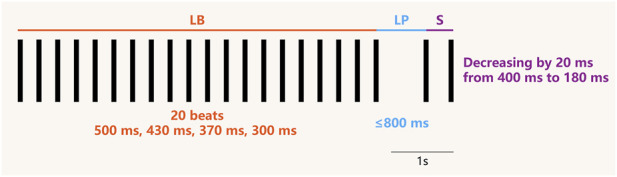
Schematic diagram of the LBLPS electrical stimulation protocol (set according to clinical standard parameters). Each black vertical line represents an extra stimulus, with the spacing between them indicating the interstimulus intervals. The LB phase consists of a series of consecutive stimuli with a fixed cycle length (dependent upon the patient’s arrhythmogenic sensitivity). The LP phase incorporates a pause slightly shorter than the sinus cycle length. The S phase involves gradually decreasing the stimulus intervals starting from 400 m until the myocardial refractory period is reached. LB, long-burst. LP, long-pause. S, short-coupled.

The current diagnostic scheme for CRDS available in animal models is the LBLPS stimulation protocol (Li et al., 2021; Sun et al., 2021; Ormerod et al., 2022; Roston et al., 2022), a combined extra stimulus composed of long-burst (LB), long-pause (LP) and short-coupled stimulus (S) (Figure 4), yet the individual implementation of each part cannot be a credible indicator for CRDS (Sun et al., 2021). Specifically speaking, long-burst, also known as burst-pacing, is applied to assess the cardiac response to rapid heart rate by delivering a series of consecutive high-frequency electrical pulse stimulation to drive the heart. Long-pause sets a relatively long interval between two successive stimuli, serving as a test for the presence of underlying autorhythmic abnormality or triggered activity in the heart after losing electrostimulation for an extended time span (Ormerod et al., 2022). And short-coupled is derived from S1S2 stimulation protocol with improvements. A sequence of stimuli at a fixed frequency (S1) can drive the heart into a steady electrophysiological state with basic cycle length, while the following single premature stimulus (S2) is imposed preceding the next basic stimulation, with the S1S2 interval shortened progressively until VAs are induced or ventricular refractory period is reached (Ripplinger et al., 2022).

**FIGURE 5:**
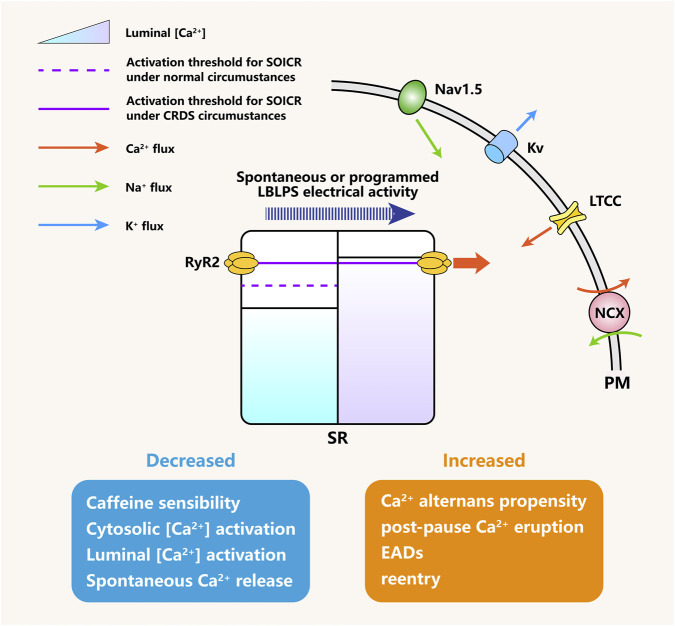
Electrophysiological remodeling and arrhythmogenesis under CRDS RyR2 LOF conditions. RyR2 LOF mutations elevate the activation threshold for SOICR. When subjected to spontaneous or programmed LBLPS electrical activity, the progressive increase in SR Ca^2+^ load culminates in the attainment of the elevated SOICR threshold, triggering an abrupt and aberrant Ca^2+^ release event. This pathological pattern of Ca^2+^ handling induces further electrophysiological remodeling and, ultimately, VAs. SOICR, store-overload-induced Ca^2+^ release. LBLPS, long-burst, long-pause and short-coupled. Nav1.5, voltage-gated sodium channel 1.5. Kv, voltage-gated potassium channel. LTCC, L-type calcium channel. NCX, sodium-calcium exchanger. PM, plasma membrane. SR, sarcoplasmic reticulum. EAD, early afterdepolarization. VA, ventricular arrhythmia.

Animal studies have indicated that the LBLPS protocol specifically induces VAs in CRDS mutants (compared with CPVT or WT) (Sun et al., 2021; Ormerod et al., 2022). All that matters is that patients with CRDS likewise experience spontaneous abnormal ECG recordings similar to those observed in LBLPS before the onset of VAs (Li et al., 2021; Sun et al., 2021; Roston et al., 2022), which seems to furnish corroborating evidence for the reliability of LBLPS testing in identifying CRDS. In terms of Ca^2+^ dynamics, it is unsurprising that the amplitude of Ca^2+^ transients decreased throughout the LB stage in intact CRDS hearts due to functionally degraded RyR2. However, an increased Ca^2+^ transient amplitude was observed after the LP stage (Sun et al., 2021; Ni et al., 2023), which could be interpreted as a post-pause eruption of SR Ca^2+^ load during the burst phase. Moreover, the extra short-coupled stimulus would trigger this enlarged Ca^2+^ wave to discharge ahead of the sinus rhythm, thereby initiating one premature systole, which corresponds to the emergence of EAD (premature excitation) monitored at the membrane potential level (Ciaccio, 2002). As discussed in the Mechanisms section, these unpredictable Ca^2+^ propagation patterns are capable of generating ectopic pacemakers in the ventricle, which can initiate VAs or even VF (Figure 5).

The heterogeneity among CRDS patients poses challenges in diagnosing and stratifying the disease. Some atypical CRDS cases exhibited unique manifestations, yet the question remains why these manifestations were only seen in specific mutants and whether they were directly linked to RyR2 LOF mutations. CRDS mouse model RyR2 I4855M^+/−^ mutants suffered from left ventricular non-compaction (LVNC), left ventricular hypertrophic cardiomyopathy and left ventricular fibrosis (Ni et al., 2023). Regarding the Ca^2+^ handling properties, I4855M^+/−^ hearts demonstrated elevated end-diastolic Ca^2+^ level, increased peak-systolic Ca^2+^ transient and prolonged Ca^2+^ transient decay time, which were distinct from the non-LVNC-associated RyR2-D4646A^+/−^ hearts (Sun et al., 2021; Ni et al., 2023). However, whether these specific Ca^2+^ handling properties are unique to the I4855M^+/−^ mutation or unmask a shared mechanism underlying RyR2 LOF-driven cardiomyopathy remains to be elucidated. Further validation across a broader spectrum of CRDS mouse models and in patient cohorts is essential to definitively establish the causal relationship between aberrant Ca^2+^ handling and the resultant cardiomyopathy phenotype. Another CRDS RyR2 LOF mutation connected to heart structure abnormalities is RyR2-V4606E^+/−^, in which carriers were afflicted with dilated cardiomyopathy (DCM) (Roston et al., 2022). The development of CRDS phenotypes characterized by cardiomyopathy, as well as the role of CRDS RyR2 LOF mutations in the progression of cardiac structural diseases, deserves further attention. These atypical manifestations highlight the diversity within CRDS and underscore the urgent need for a robust stratification system.

## 6 Therapies of CRDS

To prevent SCD caused by CRDS, implantable cardioverter-defibrillators (ICDs) have been applied in patients (Li et al., 2021; Roston et al., 2022). ICDs are commonly used as cardiac monitoring devices for patients with a history of severe arrhythmias, serving the functions of sensing abnormal heart rhythm, performing cardioversion, defibrillation and pacing, thereby correcting malignant VAs predisposed to SCD, including VT and VF (Butler et al., 2022; Crozier et al., 2023; Bragg et al., 2024). ICDs have become routinely implanted into patients’ hearts in view of their excellent efficacy and safety. The LBLPS-like abnormal electrical activities prior to the onset of VAs in CRDS patients were precisely recorded by ICDs (Li et al., 2021; Sun et al., 2021; Roston et al., 2022).

In addition to ICDs, based on the abnormal electrophysiological properties of CRDS, a variety of antiarrhythmic drugs such as quinidine, flecainide, and β-blockers have been reported to have a therapeutic or palliative effect on the symptoms of CRDS (Li et al., 2021; Sun et al., 2021; Ormerod et al., 2022; Roston et al., 2022). Of particular note is that these drugs are also extensively prescribed in the treatment of CPVT, since a large number of patients with CRDS have been misdiagnosed with CPVT caused by RyR2 GOF mutations (Roston et al., 2022). Among these drugs, flecainide stands out for its effectiveness in treating CRDS. A dosage of 20 mg/kg of flecainide in the RyR2-D4646A^+/−^ mouse model is sufficient to prevent VAs triggered by LBLPS-like electrical activity (Sun et al., 2021).

At the holistic level, the correction of RyR2 function encompasses not only the regulation of RyR2 itself, but also the elaborate network interacting with it. It is the ability to modulate the electrophysiological and mechanical function of the heart across multiple levels that accounts for the effectiveness of Na^+^ channel blockers, such as quinidine and flecainide, in the treatment of CRDS. Thoroughly grasping the role of these antiarrhythmic drugs in treating CRDS necessitates delving into their effects on the RyR2 single-channel level, along with broadening the scope of exploration to the intact heart and individual levels, as well as factoring in drug metabolism, distribution, possible side effects, and cross-reactivity between medications.

It is generally accepted that flecainide belongs to class IC antiarrhythmic drugs. However, flecainide’s antiarrhythmic role is more than a Na^+^ channel (Nav1.5) blocker, as it has binding sites on Nav1.5, NCX and RyR2 (Liu et al., 2011; Kuroda et al., 2017; Rabêlo Evangelista et al., 2021; Do and Knollmann, 2025). Flecainide possesses both activation and inhibition binding sites on RyR2 (Salvage et al., 2022). In the treatment of CPVT RyR2 GOF, the administration of flecainide resulted in a reduction in SR Ca^2+^ release, indicating an inhibitory effect on RyR2 (Kryshtal et al., 2021). Remarkably, flecainide also demonstrates effectiveness in individuals with CRDS RyR2 LOF. Such effectiveness could be attributed to the well-established Nav1.5 blocking effect of flecainide, which corrects the premature peak of the action potential and prolongs the ERP of cardiomyocytes. In this context, the activating effect of flecainide on NCX (Kuroda et al., 2017) can facilitate a more efficient and safe removal of Ca^2+^ accumulated in the cytosol after burst-pause events.

Nevertheless, the direct action of flecainide on RyR2 remains puzzling. If flecainide exerts an inhibitory effect, as it does in CPVT, then flecainide should be antiarrhythmic by suppressing the excessive release of SR Ca^2+^ stores after a long pause. However, this inhibitory effect is undoubtedly more deleterious to CRDS patients in the context of RyR2 LOF. Flecainide may also enhance RyR2 activity by binding to activation sites to resume the normal open probability (P_o_) of RyR2 and prevent Ca^2+^ overload. Therefore, beyond its efficacy, the mechanism of flecainide in the treatment of CRDS needs to be further explored.

Although the LBLPS protocol demonstrates excellent accuracy and sensitivity in diagnosing CRDS, unfortunately, its clinical application remains challenging on the grounds of the reliance on the occurrence of VAs as the criterion for a positive diagnosis, thereby further compounding the situation for cardiac patients. The spontaneous ECG activities resembling LBLPS patterns recorded in CRDS patients prior to arrhythmias warrant attention. In particular, the mechanism underlying how the latter two components, long-pause and short-coupled, drive arrhythmogenesis remains elusive. Consequently, illuminating their genesis in the context of CRDS could potentially offer insights for further improving the programmed electrical stimulation protocol, rendering it safer, non-invasive, and more feasible.

A recent multicenter collaborative study has reported that the introduction of an additional pacing stimulation to the effect of brief tachycardia followed by a subsequent pause (equivalent to LB + LP) enabled the distinction between CRDS patients and three control groups comprising patients suffering from supraventricular tachycardia (SVT), unexplained cardiac arrest (UCA), and CPVT (Ni et al., 2024). Precisely, after undergoing LBLP stimulation, the ECG recordings of the CRDS group demonstrated a prolonged QT interval with increased T-wave amplitude for the next sinus beat after the LP phase, in comparison to the three control groups (mean values). A more inspiring discovery is that 100% discrimination was achieved between the CRDS group and the other groups in terms of the change in T-wave amplitude (the variation in T-wave amplitude compared to pre-pacing values). Coupled with the benign nature of the test results, this refined LBLP protocol is supposed to be a promising diagnostic maneuver for CRDS. Accurate diagnosis, in turn, lays the foundation for exploring rational pharmacotherapy in CRDS patients.

## 7 Conclusion and perspectives

The emergence of CRDS as a distinct clinical entity associated with RyR2 LOF mutations represents a significant advancement in our understanding of the complex genetics and pathophysiology of cardiac diseases. Unlike the classic RyR2 GOF disorders such as CPVT, CRDS is characterized by RyR2 LOF mutations that impair systolic Ca^2+^ release, leading to distinct electrophysiological remodeling including enhanced I_CaL_, altered NCX activity, and action potential prolongation, which predisposes patients to EADs and arrhythmias. The identification of CRDS underscores the importance of comprehensive genetic screening in patients presenting with arrhythmias or other cardiac dysfunction, particularly when traditional diagnostic approaches fail to yield a definitive diagnosis. Notably, CRDS patients often exhibit negative EST results, necessitating distinct diagnostic tools such as the LBLPS stimulation protocol.

Our current understanding of CRDS has already yielded valuable insights into the molecular mechanisms underlying RyR2 dysfunction and its consequences on cardiac Ca^2+^ handling and contractility. However, the precise mechanisms by which diverse RyR2 mutations lead to Ca^2+^ release deficiency remain elusive, and the paradoxical relationship between mutation severity and clinical outcomes further complicates risk assessment. The description of LOF mutations in RyR2 and their relationship to impaired Ca^2+^ release provides a strong foundation for future research aimed at developing targeted therapies that specifically address the underlying defects in CRDS. Current therapeutic options are largely limited to ICDs for secondary prevention, with variable efficacy reported for antiarrhythmic drugs such as flecainide and quinidine. Further mechanistic and clinical studies are urgently needed to optimize these approaches. Despite the promising strides, numerous unanswered questions persist, inviting further research endeavors.

Currently, research on CRDS is still in its infancy, with a relatively small number of documented cases. Heterogeneity exists not only among patients with different mutation sites, but also among probands and their relatives within families sharing the same genotype (Li et al., 2021; Roston et al., 2022). Therefore, establishing a stratification system for CRDS is essential to facilitate a deeper understanding of its pathogenesis, predict disease progression, assess morbidity risk, and ultimately guide personalized treatment. Gaining insights into CRDS hinges on the continual collection of clinical data and samples from affected individuals, along with the discovery and exploration of new cases and mutation sites, all aimed at enhancing the accuracy and reliability of the CRDS stratification system.

However, relying on a single dimension of evidence may not be sufficient. Classifying CRDS patients based solely on RyR2 functional changes at the single-channel level may not accurately capture their overall clinical features. This is evident from the paradoxical finding that patients with severe RyR2 LOF mutations experienced only mild arrhythmic events, whereas patients with moderate RyR2 LOF mutations exhibited more lethal arrhythmias (Hirose et al., 2022). Nonetheless, it does not necessarily imply that there is a negative correlation between the severity of RyR2 LOF mutations and the lethality of associated arrhythmic events. Firstly, more studies on RyR2 LOF genotypes are required to substantiate this claim. Secondly, given the potential impact of severe RyR2 LOF mutations on patients, follow-up evaluations of these individuals are imperative. Further research should be dedicated to elucidating the interplay between bedside and bench manifestations of the disease, and to proposing a comprehensive and clinically applicable stratification standard for CRDS.

Furthermore, given that CRDS is currently defined by LOF mutations in RyR2 itself, an important avenue for future research will be to determine whether mutations in key RyR2 regulatory proteins, such as CASQ2, CaM, or FKBP12.6, can similarly lead to a CRDS-like phenotype, thereby expanding the spectrum of CRDS.

A major barrier to addressing these questions is the limited accessibility of primary human cardiomyocytes. Human induced pluripotent stem cell-derived cardiomyocytes (hiPSC-CMs) and cardiac organoids have emerged as powerful platforms to overcome this barrier. These patient-specific models recapitulate the native genetic background and allow functional assessment of RyR2 LOF variants under controlled conditions. For example, using hiPSC-CMs generated from a patient with a novel *MYBPC3* mutation, researchers demonstrated that RyR2 dysfunction contributes to dilated cardiomyopathy and that targeting RyR2 could rescue abnormal calcium transients (Xie et al., 2026). Although this study focused on DCM, it validates the utility of hiPSC-based approaches for dissecting RyR2-mediated pathophysiology. Extending such strategies to CRDS will enable direct validation of the pathogenicity of newly identified RyR2 LOF mutations, facilitate mechanistic studies at the cellular level, and support high-throughput screening of potential therapeutics. However, it should be acknowledged that current hiPSC-CMs and cardiac organoids remain relatively immature, often resembling neonatal cardiomyocytes and lacking key adult structural features such as T-tubules. These inherent limitations may restrict their ability to fully recapitulate mature electrophysiological responses and thus, at the present stage, may not be ideal for addressing all electrophysiological questions. Continued efforts to advance maturation protocols and engineer more adult-like cardiac tissues will be essential. Despite these challenges, integrating hiPSC-CMs and cardiac organoid models into future CRDS research holds great promise for accelerating the translation from genetic discoveries to clinical applications.

## Glossary

AF: Atrial Fibrillation
APD: Action Potential Duration
aSCD: Aborted Sudden Cardiac Death
AT: Atrial Tachycardia
CaM: calmodulin
CASQ2: calsequestrin 2
CDI: Calcium-Dependent Inactivation
CHD: Coronary Heart Disease
cIVF: Catecholaminergic Idiopathic Ventricular Fibrillation
CPVT: Catecholaminergic Polymorphic Ventricular Tachycardia
CRDS: Calcium Release Deficiency Syndrome
DAD: Delayed Afterdepolarization
DCM: Dilated Cardiomyopathy
E3DS: Exon 3 Deletion Syndrome
EAD: Early Afterdepolarization
ECC: Excitation-Contraction Coupling
ECG: Electrocardiogram
ERP: Effective Refractory Period
EST: Exercise Stress Testing
GOF: Gain-of-Function
hiPSC-CMs: Human Induced Pluripotent Stem Cell-Derived Cardiomyocytes
HF: Heart Failure
**I**_**CaL**_: L-Type Calcium Current
ICD: Implantable Cardioverter-Defibrillator
**I**_**Na**_: Fast Sodium Current
**I**_**NCX**_: Sodium-Calcium Exchange Current
IRBBB: Incomplete Right Bundle Branch Block
**I**_**to**_: Transient Outward Potassium Current
IVF: Idiopathic Ventricular Fibrillation
Kv: Voltage-Gated Potassium Channel
**LBLP**S: Long-Burst, Long-Pause and Short-Coupled
LOF: Loss-of-Function
LQTS: Long-QT Syndrome
LTCC: L-type Calcium Channel
LVNC: Left Ventricular Non-Compaction
Nav1.5: Voltage-Gated Sodium Channel 1.5
NCX: Sodium-Calcium Exchanger
NSVT: Non-Sustained Ventricular Tachycardia
PM: Plasma Membrane
**P**_**o**_: Open Probability
PVC: Premature Ventricular Complex
RyR2: Cardiac Ryanodine Receptor
SCA: Sudden Cardiac Arrest
SCD: Sudden Cardiac Death
scPVC: Short-Coupled Premature Ventricular Contract
scTdP: Short-Coupled Variant of Torsades de Pointes
SERCA: Sarcoplasmic/Endoplasmic Reticulum Calcium ATPase
SOICR: Store-Overload-Induced Calcium Release
SR: Sarcoplasmic Reticulum
SVT: Supraventricular Tachycardia
TnC: Troponin C
UCA: Unexplained Cardiac Arrest
VA: Ventricular Arrhythmia
VF: Ventricular Fibrillation
VT: Ventricular Tachycardia
